# Molecular docking analysis of AGTR1 antagonists

**DOI:** 10.6026/97320630019284

**Published:** 2023-03-31

**Authors:** Hussam Aly Sayed Murad, Misbahuddi M Rafeeq, Saleh Mudawi Alqahtani, Bodour S. Rajab, Saad Alghamdi, Samah J. Almehmadi, Qamre Alam

**Affiliations:** 1 Department of Pharmacology, Faculty of Medicine, Rabigh, King Abdulaziz University, Jeddah, Saudi Arabia; 2 King Faisal Medical City, Medical laboratory department, Abha 62529, Saudi Arabia; 3 Laboratory Medicine Department, Faculty of Applied Medical Sciences, Umm Al-Qura University, Makkah, Saudi Arabia; 4 Molecular Genomics and Precision Medicine, ExpressMed Laboratories, Zinj, Bahrain

**Keywords:** Cardiovascular disease, natural compounds, AGTR1, drug-likeness

## Abstract

Cardiovascular diseases (CVDs) are the leading cause of death and morbidity globally. The renin-angiotensin system is an important regulatory system for maintaining cardiovascular and renal function. Therefore, angiotensin-converting enzyme inhibitors and angiotensin receptor blockers have emerged as first-line treatments for conditions such as hypertension and heart failure. Currently available synthetic medications used to treat various CVDs have been linked with various adverse effects. Therefore, this study focuses on targeting type-1 angiotensin II receptor (AGTR1) by natural compounds. The ZINC database natural compounds and standard AGTR1 inhibitors have been screened against the AGTR1 active site. The results showed that five compounds, namely ZINC85625504, ZINC62001623, ZINC70666587, ZINC06624086, and ZINC95486187, had similar binding energies to established AGTR1 inhibitors. These compounds were found to interact with crucial AGTR1 residues, indicating their potential as AGTR1 inhibitors. Moreover, the hit compounds demonstrated favorable drug-like characteristics and warrant further investigation for their potential use in managing CVD.

## Background:

Cardiovascular diseases (CVDs) are the primary cause of mortality and morbidity worldwide [1]. The primary drivers of the rise in CVDs are urbanization and lifestyle changes. CVD mortality, mostly due to ischemic heart disease and stroke, has been declining in high-income nations (Europe, North America, and Australia) since the late twentieth century, and the trend is expected to continue, but the pace of decrease has lately slowed. Nonetheless, the incidence of CVDs is anticipated to rise owing to the prolonged lifespan of individuals with CVDs, while the absolute count of CVD fatalities will also increase due to population aging. Under the assumption of stable major cardiovascular risk factors, a significant increase in the prevalence of heart disease or stroke among middle-aged individuals is expected in the majority of countries, resulting in a significant number of CVD fatalities in the 35-64 age group over the next three decades [2, 3].

**Figure 1:**
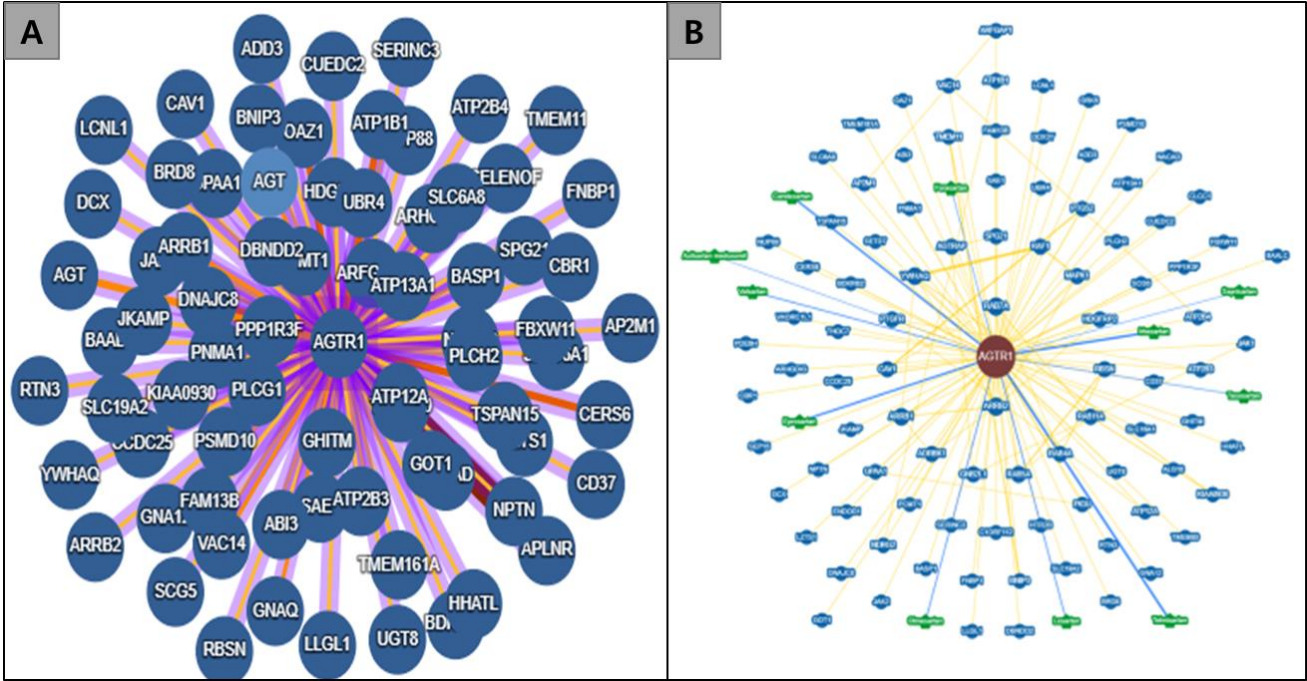
Interaction of AGTR1 with other proteins. Predicted by IntAct database (A), and BioGRID database (B).

The renin-angiotensin system is an important regulatory system for maintaining cardiovascular and renal function. Therefore, angiotensin-converting enzyme inhibitors and angiotensin receptor blockers have emerged as first-line treatments for conditions such as hypertension and heart failure [4]. Here in this study, we focus on targeting type-1 angiotensin II receptor (AGTR1) among the several potential druggable targets. The activation of the AGTR1 receptor by angiotensin II results in vasoconstriction, sodium retention, and oxidative stress, all of which contribute to the development of hypertension, heart failure, and other CVDs [5]. AGTR1 blockers are a class of drugs that inhibit the activation of AGTR1 selectively, thereby reducing the negative effects of angiotensin II. These medications are commonly used to treat hypertension, heart failure, and other CVDs. Multiple studies have demonstrated that targeting AGTR1 can reduce the incidence of cardiovascular events. Targeting the AGTR1 with drugs such as losartan, valsartan, and irbesartan has been demonstrated to be an effective CVD management strategy [6]. Shreds of evidence from literature and protein-protein interaction analysis of AGTR1 with other proteins show that it interacts with several proteins. Numerous protein-protein interaction databases show how AGTR1 interacts with other proteins. For instance, the IntAct database, an open-source molecular interaction database, predicts interactions of AGTR1 with 92 proteins using data either selected from the literature or through direct data depositions [7]. The BioGRID database, a freely accessible repository for genetic and protein interaction information from model organisms and people, has 104 interactors [8] (Figure 1).

There are several known inhibitors/blockers of AGTR1 for example; Losartan, Valsartan, Irbesartan, Candesartan, Telmisartan, medoxomil, and Azilsartan medoxomil . Several currently available synthetic medications used to treat various CVDs have been linked with a number of adverse effects. Consequently, natural compounds have gained popularity in the modern era due to their low cost, easy availability, high effectiveness, and fewer side effects.

## Methodology:

## Preparation of standard inhibitors and natural compounds library preparation:

This study utilized a carefully selected collection of natural compounds sourced from the ZINC database. The compounds were filtered using the 'Lipinski and Veber Rule' and were chosen to have molecular weights within the range of 300 to 500. The resulting curated library contained a total of 350 compounds. These compounds were minimized and prepared in pdbqt format for further docking analysis. ZD7 (co-crystal ligand), candesartan, losartan, and valsartan, all well-known inhibitors of AGTR1, were prepared for docking analysis as a positive control for the screening.

## Target protein (AGTR1) preparation for docking analysis:

The 3D structure of AGTR1 was obtained from the RCSB PDB (PDB ID: 4YAY) [9]. The structure is asymmetric and monomeric having a co-crystallized ligand ZD7. Water, heteroatoms, and co-crystallized ligands were eliminated, and the protein was cleaned and processed with DS before being saved as a '.pdb' file for virtual screening (VS)/docking purposes.

## Structure based virtual screening:

The PyRx program was used to perform VS of prepared natural compounds and standard inhibitors against the active site of prepared target proteins [10]. The docked complexes were subsequently assessed using DS Visualizer and Pymol, and the ideal conformation was determined based on the lowest binding energy.

## Physicochemical properties, ADME, and toxicity prediction:

Physicochemical properties, ADME, and toxicity estimation were predicted for the top 20 screened compounds. The physicochemical characteristics and pharmacokinetic profile of a therapeutic substance, which includes absorption, distribution, metabolism, excretion, and toxicity (ADMET), are crucial in determining its pharmacodynamic properties. The "ADMET Descriptor" module in DS was used to calculate the ADMET characteristics of the phytochemicals. The "TOPKAT" module in DS was used to evaluate toxicity.

## Results and Discussion:

**Figure 2:**
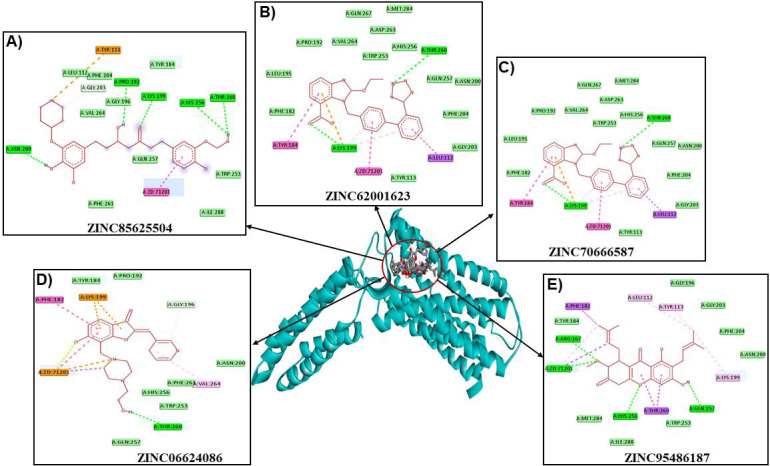
Interaction of best 5 hits with the active site residues of AGTR1.

In this study, we selected four positive controls namely, ZD7 (co-crystal ligand), candesartan, losartan, and valsartan which are widely known inhibitors of AGTR1. A curated database of 350 natural compounds obtained from the ZINC database was screened against the active site of AGTR1. These compounds were filtered using the 'Lipinski and Veber Rule' and had molecular weights within the range of 300 to 500. A grid of XYZ axes was set up for the molecular docking-based VS (X=-16.087, Y=9.764, and Z= 41.290). The screening results revealed that several compounds had higher binding energies when compared to control compounds (Table 1), but after in-depth analysis and visualization of the docked complexes' 2D and 3D interactions, 16 compounds demonstrated more effective binding in terms of interaction with critical AGTR1 residues such as THR260. Here we discussed and demonstrated the top 5 natural compounds as potential hits. ZINC85625504 interacted with Tyr113, Tyr184, Leu112, Phe204, Pro192, Gly203, Gly196, Lys199, Val264, Lys199, His256, Thr260, Trp253, Gln257, Ile288, Phe261, and Asn200 residues of AGTR1. Residues Pro192, Lys199, His256, Thr260, and Asn200 H-bonded with ZINC85625504 (Figure 2A). ZINC62001623 bind with Tyr184, Phe182, Leu195, Pro192, Val264, Gln267, Met284, Asp263, Trp253, His256, Thr260, Gln257, Asn200, Phe204, Gly203, Leu112, Tyr113, and Lys199 residues of AGTR1. Residues Thr260, and Lys199 H-bonded with ZINC62001623 (Figure 2B). ZINC70666587 interacted Tyr184, Phe182, Leu195, Pro192, Val264, Gln267, Met284, Asp263, His256, Trp253, Thr260, Gln257, Asn200, Phe204, Gly203, Leu112, Tyr113, and Lys199 residues of AGTR1. ZINC70666587 H-bonded with Lys199, and Thr260 residues of AGTR1 (Figure 2C). In addition, ZINC06624086 interacted with Phe182, Lys199, Tyr184, Pro192, Gly196, Asn200, Val264, His256, Trp253, Thr260, nad Gln257 residues of AGTR1. Residue Thr260 H-bonded with the ZINC06624086 (Figure 2D). ZINC95486187 bind with Phe182, Tyr184, Arg167, Met284, His256, Thr260, Ile288, Trp253, Gln257, Lys199, Asn200, Phe204, Gly203, Gly196, Tyr113, and Leu112 residues of AGTR1. Residues Arg167, His256, and Gln257 H-bonded with ZINC95486187 (Figure 2E). The residues Leu112, Lys199, Asn200, Trp253, His256, Gln257, and Thr260 in AGTR1 have been identified as crucial for binding with inhibitors [11]. It is noteworthy that the compounds (ZINC85625504, ZINC62001623, ZINC70666587, ZINC06624086, and ZINC95486187) have been observed to bind with these AGTR1 residues.

**Figure 3:**
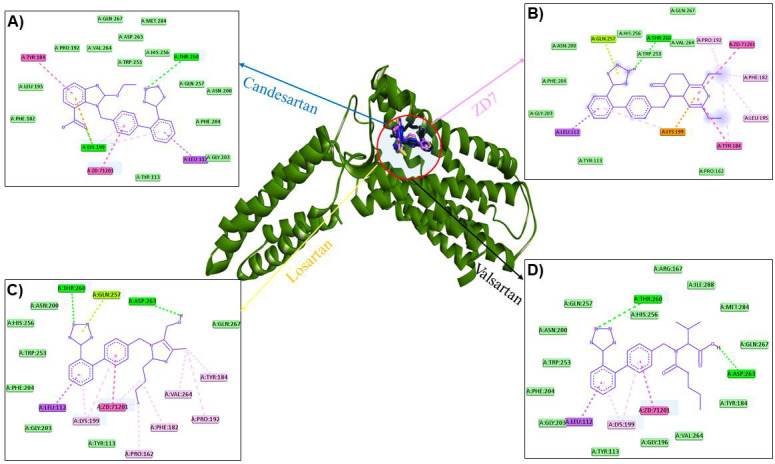
Interaction of positive controls with the active site residues of AGTR1.

Furthermore, the interaction profile of positive controls with AGTR1 protein was analyzed. Candesartan interacted with Thr184, Pro192, Val264, Gln267, Asp263, Met284, Trp253, His256, Thr260, Gln257, Asn200, Phe204, Gly203, Leu112, Tyr113, Lys199, Phe182, and Leu195 residues of AGTR1 (Figure 3A); while Leu112, Gly203, Phe204, Asn200, Gln257, His256, Thr260, Trp253, Val264, Pro192, Gln267, Phe182, Leu195, Tyr184, Pro162, Lys199, and Tyr residues interacted with ZD7 (Figure 3B). Losartan interacted with Thr260, Gln257, Asp263, Gln267, Tyr184, Val264, Pro192, Phe182, Pro162, Tyr113, Lys199, Leu112, Gly203, Phe204, Trp253, His256, and Asn200 residues of AGTR1 (Figure 3C). In addition, valsartan interacted with Thr260, His256, Arg167, Ile288, Met2 84, Gln267, Asp263, Tyr184, Val264, Gly196, Lys199, Tyr113, Leu112, Gly203, Phe204, Trp253, Asn200, and Gln257 residues of AGTR1 (Figure 3D). It is worth noting that Thr260 was identified as the common H-bonded residue of the AGTR1 protein with the hits (ZINC85625504, ZINC62001623, ZINC70666587, and ZINC06624086) as well as the positive controls (candesartan, ZD7, losartan, and valsartan)(Figure 2A-D & Figgure 3A-D).

The physicochemical, ADME, and toxicological features of the top 20 natural compounds were investigated. Since the screened library had already been filtered by the 'Lipinski and Veber Rule' and had molecular weights ranging from 300 to 500, most of the screened compounds were nontoxic. According to TOPKAT and ADMET forecasts, few of the chemicals are carcinogenic and the majority of the compounds are not mutagenic (Table 2).

## Conclusion:

This study utilized computational methods including structure-based VS, ADME, and interaction analysis to identify compounds (ZINC85625504, ZINC62001623, ZINC70666587, ZINC06624086, and ZINC95486187) that can bind to the AGTR1 protein, a target for therapies for CVDs. These compounds also exhibited favorable drug-like characteristics, indicating their potential as candidates for treating CVDs.

## Funding Statement:

This research work was funded by Institutional Fund Projects under grant no. (IFPIP:64-828-1443). The authors gratefully acknowledge technical and financial support provided by the Ministry of Education and King Abdulaziz University, DSR, Jeddah, Saudi Arabia.

## Figures and Tables

**Table 1:** Binding energy of top 20 screened compounds and positive controls.

**Ligand**	**Binding energy (kcal/mol)**
ZINC06624086	-11.2
ZINC95486187	-10.6
ZINC19804810	-10.3
ZD7	-10.2
Candesartan	-10.1
ZINC62001623	-10.1
ZINC70666587	-9.9
ZINC85625504	-9.9
ZINC06624236	-9.9
ZINC02109240	-9.9
ZINC96113966	-9.8
ZINC02109241	-9.8
ZINC02145358	-9.7
ZINC32502206	-9.7
ZINC02119331	-9.7
ZINC20611818	-9.6
Losartan	-9.5
ZINC08918025	-9.5
ZINC32124198	-9.4
ZINC32124036	-9.1
Valsartan	-8.9
ZINC19804812	-8.9
ZINC32124056	-8.9
ZINC20760145	-8.9

**Table 2:** Physicochemical, and ADME properties of top 20 hits. (HA: Hydrogen bond acceptor, HD: Hydrogen bond donor, PSA: Polar Surface Area)

Compound name	HA	HD	Mol Weight	ALogP	PSA	CYP2D6	CYP2D6	CYP2D6	Hepatotoxic				PPB			TOPKAT Ames_		
						CYP2D6	Applicability #MD	Applicability Mdpvalue	Hepatotoxic	Applicability	Applicability Mdpvalue	PPB	Applicability #MD	Applicability #Mdpvalue	Probab	Enrich	Score
ZINC02109240	7	1	475.579	3.366	71.11	-2.89209	16.6607	2.54E-08	-5.58453	13.8603	2.13E-08	0.618968	15.7424	8.44E-09	0.358954	0.642859	-10.5502
ZINC02109241	7	1	475.579	3.366	71.11	-2.89209	16.6607	2.54E-08	-5.58453	13.8603	2.13E-08	0.618968	15.7424	8.44E-09	0.358954	0.642859	-10.5502
ZINC02119331	4	0	412.477	6.49	52.6	2.02863	14.7537	3.86E-06	-1.8393	12.6769	8.42E-06	5.58852	13.4863	0.000929062	0.48416	0.867092	-7.87028
ZINC02145358	7	4	384.449	-0.604	86.47	-2.84067	18.4924	1.91E-10	-2.77142	11.7359	0.000462	-8.30574	15.1783	2.45E-07	0.411286	0.736582	-9.44712
ZINC06624086	7	4	384.449	-1.116	86.47	-1.42279	19.1742	3.13E-11	-3.9077	13.0981	1.12E-06	-7.49713	14.6371	4.70E-06	0.291756	0.522512	-11.9897
ZINC06624236	7	4	384.449	-1.31	86.47	-1.86548	19.4379	1.56E-11	-2.65499	11.8431	0.000304	-9.07947	13.7254	0.000348031	0.376731	0.674696	-10.1758
ZINC08918025	6	1	475.576	4.503	76.07	-5.70885	16.7292	2.12E-08	-2.18298	13.747	3.93E-08	3.1818	15.2969	1.24E-07	0.039914	0.0714829	-21.3897
ZINC19804810	4	0	453.572	7.977	38.77	2.33903	17.6859	1.64E-09	0.290432	15.8135	1.80E-13	5.23401	15.3026	1.20E-07	0.389888	0.698258	-9.89879
ZINC19804812	5	1	453.555	5.612	76.9	-6.34672	14.5658	6.27E-06	-3.63965	14.6233	2.82E-10	4.74442	12.372	0.0380477	0.597402	1.0699	-5.10531
ZINC20611818	3	0	382.451	6.315	35.53	0.04005	10.9877	0.022645	-3.49772	12.7304	6.56E-06	7.77075	12.5819	0.0211427	0.130833	0.234312	-16.1644
ZINC20760145	6	0	382.453	3.103	62.99	-6.98662	14.7932	3.49E-06	1.61334	13.1045	1.09E-06	2.98716	16.3988	1.18E-10	0.323781	0.579866	-11.2964
ZINC32124198	5	1	453.555	5.612	76.9	-6.34672	14.5658	6.27E-06	-3.63965	14.6233	2.82E-10	4.74442	12.372	0.0380477	0.597402	1.0699	-5.10531
ZINC32502206	7	3	479.591	4.159	111.3	-1.96007	22.7936	2.70E-15	-2.41679	16.0229	4.63E-14	1.73866	17.7813	4.78E-15	0.483466	0.865849	-7.88576
ZINC62001623	7	3	453.554	2.024	124.04	-5.70048	14.6924	4.53E-06	-6.32363	10.042	0.082154	-12.151	17.413	8.11E-14	0.0487713	0.0873456	-19.9974
ZINC70666587	7	3	453.554	2.024	124.04	-5.70048	14.6924	4.53E-06	-6.32363	10.042	0.082154	-12.151	17.413	8.11E-14	0.0487713	0.0873456	-19.9974
ZINC95486187	6	2	412.476	4.352	93.06	-0.30927	17.1182	7.49E-09	-0.85309	14.6296	2.72E-10	0.280967	13.0185	0.00526403	0.165383	0.296188	-15.093
ZINC96113966	6	3	455.586	1.612	95.86	-4.27437	13.4842	9.49E-05	-9.07579	12.4892	1.98E-05	-0.56175	12.5752	0.021559	7.51E-06	1.34E-05	-41.651
ZINC32124056	5	1	453.555	5.612	76.9	-6.34672	14.5658	6.27E-06	-3.63965	14.6233	2.82E-10	4.74442	12.372	0.0380477	0.597402	1.0699	-5.10531
ZINC32124036	5	1	453.555	5.612	76.9	-6.34672	14.5658	6.27E-06	-3.63965	14.6233	2.82E-10	4.74442	12.372	0.0380477	0.597402	1.0699	-5.10531

## References

[1] Roth GA (2020). J Am Coll Cardiol.

[2] Roth GA (2017). J Am Coll Cardiol.

[3] Beaglehole R (2011). Lancet.

[4] Ma TK (2010). Br J Pharmacol.

[5] Kucmierz J (2021). Int J Mol Sci.

[6] Sriram K (2020). Proceedings of the National Academy of Sciences of the United States of America.

[7] Orchard S (2014). Nucleic acids research.

[8] Oughtred R (2016). Cold Spring Harbor Protocols.

[9] Zhang H (2015). Cell.

[10] Trott O, Olson AJ (2010). J Comput Chem.

[11] Zhang H (2015). J Biol Chem.

